# Interaction of Mean Temperature and Daily Fluctuation Influences Dengue Incidence in Dhaka, Bangladesh

**DOI:** 10.1371/journal.pntd.0003901

**Published:** 2015-07-10

**Authors:** Sifat Sharmin, Kathryn Glass, Elvina Viennet, David Harley

**Affiliations:** National Centre for Epidemiology and Population Health, Research School of Population Health, The Australian National University, Canberra, Australian Capital Territory, Australia; Armed Forces Health Surveillance Center, UNITED STATES

## Abstract

Local weather influences the transmission of the dengue virus. Most studies analyzing the relationship between dengue and climate are based on relatively coarse aggregate measures such as mean temperature. Here, we include both mean temperature and daily fluctuations in temperature in modelling dengue transmission in Dhaka, the capital of Bangladesh. We used a negative binomial generalized linear model, adjusted for rainfall, anomalies in sea surface temperature (an index for El Niño-Southern Oscillation), population density, the number of dengue cases in the previous month, and the long term temporal trend in dengue incidence. In addition to the significant associations of mean temperature and temperature fluctuation with dengue incidence, we found interaction of mean and temperature fluctuation significantly influences disease transmission at a lag of one month. High mean temperature with low fluctuation increases dengue incidence one month later. Besides temperature, dengue incidence was also influenced by sea surface temperature anomalies in the current and previous month, presumably as a consequence of concomitant anomalies in the annual rainfall cycle. Population density exerted a significant positive influence on dengue incidence indicating increasing risk of dengue in over-populated Dhaka. Understanding these complex relationships between climate, population, and dengue incidence will help inform outbreak prediction and control.

## Author Summary

The sensitivity of mosquito vector and dengue virus biology to diurnal temperature variability has been established, but this study is the first analyzing these relations with dengue occurrence. We show that Dhaka’s tropical hot monsoon climate and small variation in daily temperature enhance dengue transmission one month later. Large-scale climatic events like El Niño-Southern Oscillation and increasing population density of Dhaka also increase incidence. Our results therefore enable us to accurately estimate dengue transmission dynamics in densely populated areas that are also vulnerable to global warming by considering diurnal variability. Our approach reduces the chance of overestimating the effect of increasing temperature on dengue transmission intensity with the ultimate goal of outbreak prediction and control.

## Introduction

Dengue virus (DENV) [1] transmission occurs in more than 100 countries; however, the burden of dengue is not evenly distributed. Approximately half of the global population at risk of acquiring dengue infection resides in the South-East Asia Region of the World Health Organization [2], a region characterized by strong seasonal weather variation and heavy monsoon rainfall. This reflects the influence of local weather, particularly temperature and rainfall, on the transmission of DENV by *Aedes* mosquitoes. Higher temperature, for example, shortens mosquito development time [3], increases the frequency of blood feeding presumably by decreasing body size [4, 5], and reduces the extrinsic incubation period of DENV within mosquitoes [6]. However, transmission of DENV is influenced not only by average temperature, but also by diurnal temperature range (DTR, the difference between daily maximum and minimum temperature). Temperature-dependent empirical and mathematical experiments show that temperature fluctuation influences vectorial capacity of *Aedes aegypti*, the principal mosquito vector of DENV, via biting rate, DENV transmission probability, extrinsic incubation period, and vector mortality rate [7–10]. At high mean temperatures, vectorial capacity increases with narrow daily temperature variation [7–9]. At low mean temperatures, the relationship between DTR and vectorial capacity is reversed [7–10]. Temperatures above 30°C reduce survival of adult *Ae*. *aegypti* [11] as does either very low or very high rainfall [12]. The positive relationship between rainfall and dengue incidence has been observed in several locations [13–15]. Seemingly paradoxical is the observation that the incidence of dengue increases in the dry season in some locations [16]. Large scale climatic events, such as the Southern Oscillation, resulting from the interplay of large scale ocean and atmospheric circulation processes in the equatorial Pacific Ocean have been identified as a remote driver of inter-annual weather variability across the globe. The warm and cold phases of the Southern Oscillation, El Niño and La Niña, respectively, are known to influence local temperature and rainfall and hence year-to-year variations in dengue incidence [13, 17, 18]. Socio-demographic and economic factors also influence dengue incidence. While the population at risk of dengue is likely to rise with increasing population, economic development would be expected to reduce risk [19].

Bangladesh, a member country of the World Health Organization South-East Asia Region experienced its first epidemic of dengue fever in 2000 after more than three decades of sporadic dengue [20]. Dengue is highly seasonal in Bangladesh with increased incidence during the monsoon. From 2000 to 2009, cases have been reported from 29 of the 64 Bangladeshi districts, with around 91.0% from the capital, Dhaka [21]. Since 2010 very few cases have been notified from districts other than Dhaka [21] presumably because of a change in reporting criteria requiring confirmatory laboratory diagnosis.

Studies of dengue in Bangladesh before ours have not considered daily temperature variation [22, 23]. We present an analysis of the influence of daily temperature variation on the transmission of dengue adjusted for rainfall and population density, using a monthly dengue case time-series over 10 years from Dhaka. We also considered anomalies in sea surface temperature (SSTA), an index for El Niño-Southern Oscillation (ENSO) that is associated with extreme weather in Bangladesh and has not been included in other studies. Analyses such as ours are critical for understanding the associations between weather, population, and dengue incidence and will allow the development of a reliable dengue early warning system.

## Materials and Methods

### Ethics Statement

The study was approved by The Australian National University Human Research Ethics Committee. The national surveillance data of dengue fever cases was anonymized.

### Study Area

Dhaka district, comprising Dhaka Metropolitan area (DMA) and adjacent sub-districts, is a 1,464 km^2^ area near the center of Bangladesh. Of the 64 districts this is the most densely populated, currently with 8,229 people per square kilometer. Over the years 2001 to 2011, there was a 41.0% increase in the population density of Dhaka [24]. More than 37.0% of the population of DMA live in slums with a population density of 220,246 people per square kilometer [25]. Slums have no access to piped water and temporary containers like drums and earthen jars are commonly used to store water in which *Ae*. *aegypti* lays eggs [26]. Inadequate supplies of piped water and an absence of proper waste management in most locations of Dhaka result in abundant indoor and outdoor mosquito breeding sites. Both *Ae*. *aegypti* and *Aedes albopictus*, the latter a secondary vector of dengue, were observed in Dhaka during the 2000 epidemic [27]. Unscreened doors and windows permit mosquito entry to dwellings.

Dhaka has a hot and humid tropical climate, with an average temperature of approximately 25°C, which nearly always permits mosquito development and DENV transmission. Rainfall is highly seasonal, with the wettest period (June to September) occurring during the warmest months. About 80.0% of the annual rainfall of 2,000 mm falls during the monsoon. Rainfall in Bangladesh is partly influenced by the Southern Oscillation with El Niño years usually associated with less than average monsoon rainfall while the opposite has been observed in La Niña years. However, the influence of the Southern Oscillation on monsoon rainfall is not linear and is inconsistent, as observed in the moderate El Niño years causing flooding while some La Niña events during the monsoon preceded by El Niño are associated with reduced monsoon rainfall in Bangladesh [28, 29].

### Data Set

Monthly dengue cases for Dhaka district, from January 2000 to December 2009, were obtained from the Directorate General of Health Services. This time period was chosen to avoid the influence of the change in reporting practice started in 2010.

The daily maximum, minimum, and mean temperatures (°C), relative humidity (%), and rainfall (mm) data for Dhaka were collected from the Bangladesh Meteorological Department. A single missing value for maximum temperature was replaced by linear interpolation. Diurnal temperature range was derived as the difference between maximum and minimum daily temperature. Monthly means of these climatic variables were calculated from the daily records. A monthly time series of SSTA over the Niño 3.4 region was obtained from the United States National Oceanic and Atmospheric Administration Climate Prediction Center (http://www.cpc.ncep.noaa.gov/data/indices/ersst3b.nino.mth.81-10.ascii). The Niño 3.4 index was used because of its correlation with Indian Ocean region monsoon rainfall. An increase (decrease) of >0.5°C (<-0.5°C) in three-month moving average of SSTA is referred to as an El Niño (a La Niña) event.

Population estimates were extracted from the 1991, 2001, and 2011 census data (there was no census taken between these years) of the Bangladesh Bureau of Statistics. Linear interpolation was used to calculate the monthly population for each of the years between 2000 and 2009. The population density (people/km^2^) for Dhaka was estimated by dividing the district population size by the area (km^2^).

### Analyses

To examine temporal patterns over the study period, monthly dengue cases and climatic averages were plotted over the 10-year period. To display seasonal patterns, monthly averages of mean temperature, DTR, and rainfall, and monthly numbers of total dengue cases over the 10 years were aggregated and plotted by month.

Overall correlation between dengue cases and climatic variables (mean monthly temperature, mean monthly DTR, mean monthly relative humidity, mean monthly rainfall, and monthly SSTA) were examined using Spearman's rank correlation test. To avoid multicolinearity arising from correlated variables, the final set of candidate variables was restricted to those with pair-wise correlations of ≤0.8.

Cross-correlation functions of dengue cases with each of the climatic variables were then estimated to investigate their lagged effects on dengue incidence (*p*≤0.05). Time lags were included to account for the influence of climatic variables on the development, maturation, and survival of the vector (*Aedes* mosquitoes) as well as the extrinsic incubation period of DENV in the vector and the intrinsic incubation period in the human host. Lags of up to three months were considered for all weather variables, with SSTA also considered at a lag of four months.

The counts of dengue cases were then fitted by a generalized linear model (GLM) with negative binomial distribution to allow for overdispersion in dengue counts. The population of Dhaka was added as an offset to the model on a logarithmic scale to adjust for population size. Population density was also included in the model to account for the potential influences of associated socio-demographic changes on dengue transmission in Dhaka.

An indicator variable for outbreak months was added to prevent occasional extreme values from distorting the analyses. A month with the number of dengue cases exceeding the 10-year mean plus two standard deviations was defined as an outbreak month. To account for the long term trend in dengue incidence over time, an indicator variable for year was incorporated in the model. An autoregressive term at order 1 was also included to allow for autocorrelation in monthly numbers of dengue cases. To determine whether seasonal variation had any influence on dengue incidence, a categorical variable for winter (December–February), pre-monsoon (March–May), monsoon (June–September), and post-monsoon (October–November) was also considered.

The analyses were performed using STATA 13.1 (StataCorp., Texas, USA) and figures were drawn using RStudio (R development Core Team, 2015).

## Results

**Fig 1:**
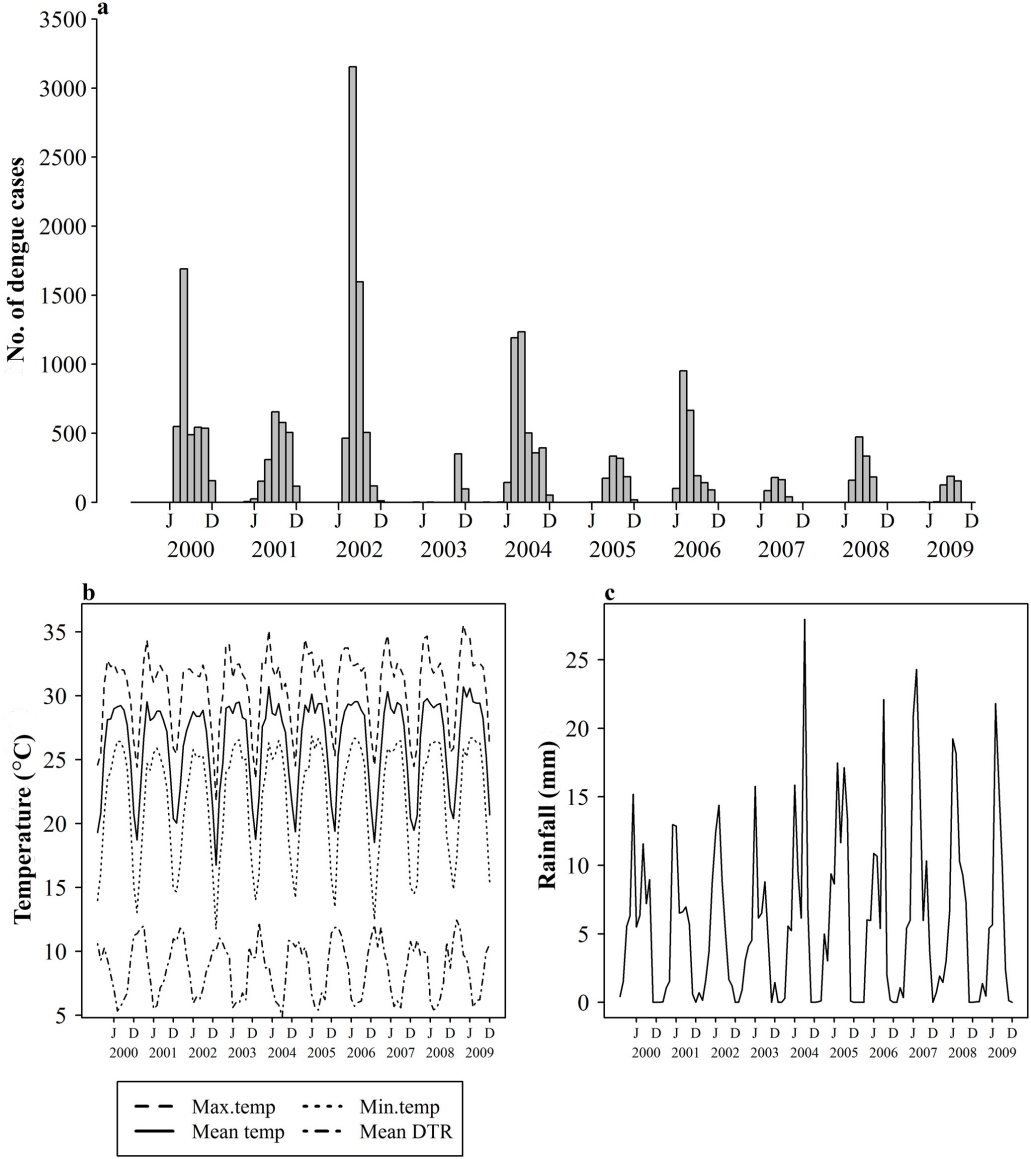
Time series of dengue cases and meteorological variables from Dhaka (2000–2009). a) Monthly dengue cases b) Average maximum, mean, and minimum monthly temperatures (°C) and mean monthly DTR (°C) (top to bottom) c) Mean monthly rainfall (mm).

**Fig 2:**
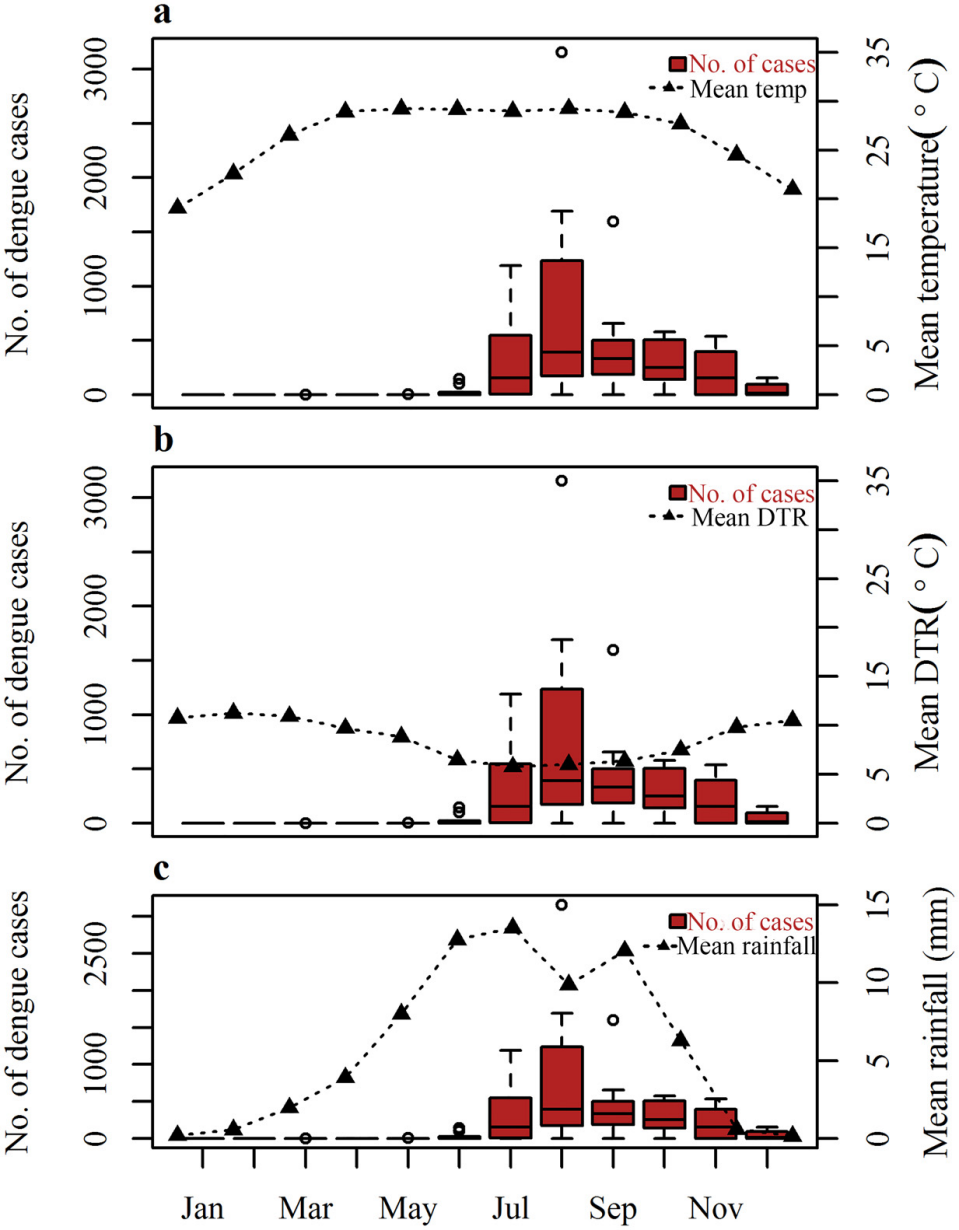
Boxplots of the monthly distribution of total dengue cases during 2000–2009 and a) Mean monthly temperature (°C) b) Mean monthly DTR (°C) c) Mean monthly rainfall (mm) for Dhaka, averaged for each month over 2000–2009. The boxplots display the median value as a line inside the box, the 25th and 75th percentile by the box, the range of values by the whiskers outside the box, and potential outliers by unfilled circles.

Inter-annual and seasonal variations for dengue and weather over the period 2000–2009 are presented (Figs 1 and 2). The number of dengue cases during winter is low and starts to increase from June (Fig 2) with the advent of the monsoon with considerable annual variation (Fig 1). The peak comes one month after the initial rainfall peak in July and starts declining afterwards. Temperature reaches its peak in April and plateaus until October when it drops (Fig 2).

Because of the high correlation with mean temperature and DTR, relative humidity was excluded at the initial stage of model formulation. Consideration of both temperature and rainfall was, however, expected to minimize the potential confounding effect of relative humidity on dengue incidence. The categorical variable for season was also subsequently removed because it did not improve model fit. Therefore, the model finally fitted is as follows:

$$y_{t}\sim NegBin\left(\mu_{t},\theta \right)$$

$$\begin{array}{l} \text{log}(\mu_{t})=\alpha +\sum_{j=0}^{3}\beta_{1j}T_{jt}+\sum_{j=0}^{3}\beta_{2j}DTR_{jt}+\sum_{j=0}^{3}\beta_{3j}(T_{jt}\times DTR_{jt})+\sum_{j=0}^{3}\beta_{4j}R_{jt}\\ +\sum_{j=0}^{4}\beta_{5j}SSTA_{jt}+\beta_{6}Popden_{t}+outbreak+year+y_{t-1}\\ +\text{log}(Population)+\varepsilon_{t} \end{array}$$ (1)

where *y*_*t*_ is the dengue count in Dhaka in month *t* (*t* = 1,…,120); μ_*t*_ is the corresponding mean dengue count; *T*, *DTR*, *R*, and *SSTA* are the mean monthly temperature (°C), mean monthly diurnal temperature range (°C), mean monthly rainfall (mm), and monthly sea surface temperature anomaly respectively; (*T*×*DTR*) represents the interaction between mean monthly temperature and mean monthly DTR; j = 0,…,4 represent the time lag periods in months; *outbreak* is the categorical variable for outbreak months; *year* represents time trend; *y*_t-1_ is the dengue count of previous month; and ε_*t*_ is the error term.

Table 1 shows estimates of the significant covariates from model (1). Mean temperature, DTR, and the interaction between these two variables are all significant predictors of dengue incidence at a lag of one month. However, the opposing directions of main and interaction effects indicate a negative synergy between mean temperature and DTR. Therefore, dengue incidence increases with higher temperature and lower DTR or lower temperature and higher DTR in the previous month but decreases when both are either high or low.

**Table 1:** Parameter estimates for significant covariates.

**Variable**	**Coefficient estimate**	**95% Confidence Interval**
Temperature lag 1	6.07**	3.38, 8.67
DTR lag 1	15.57**	8.03, 22.85
(Temperature*DTR) lag 1	-0.56**	-0.81, -0.29
Rainfall lag 1	0.14**	0.04, 0.23
Rainfall lag 2	0.17**	0.07, 0.28
SSTA lag 0	-3.37**	-5.22, -1.51
SSTA lag 1	2.63*	0.16, 5.09
Popden	0.05*	0.01, 0.09

* Significant at *p*<0.05,

** Significant at *p*<0.01.

Rainfall at lag one and two months was found to be positively associated with dengue incidence, suggesting that increased incidence of dengue in a given month is associated with higher rainfall during the previous two months.

The negative effect of SSTA on dengue incidence at lag zero month indicates that the incidence goes up with increasing negative values of the SSTA in the current month, while the inverse relationship was observed at lag of one month.

Increasing population density, as anticipated, increases dengue incidence.

**Fig 3:**
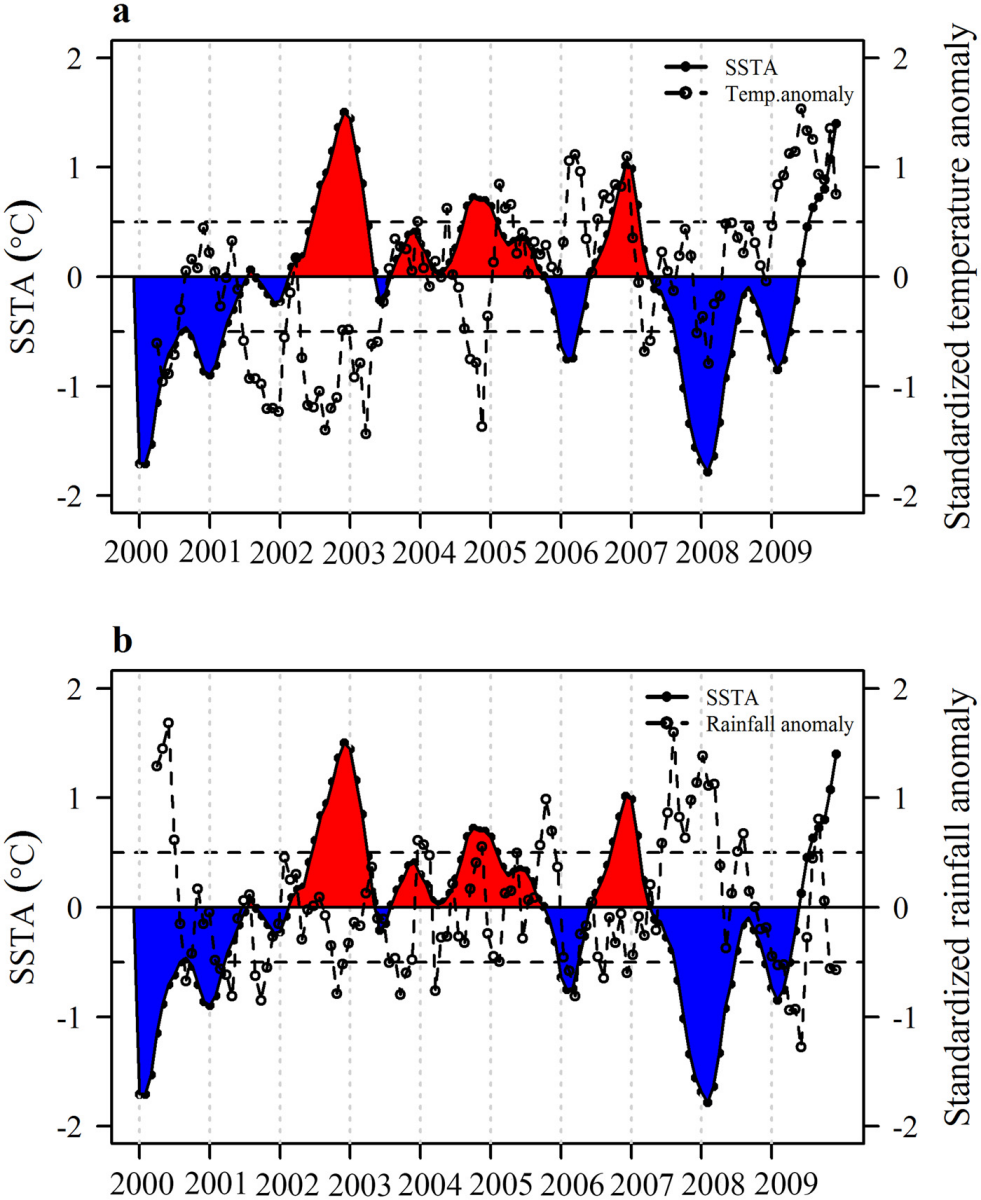
Three-month moving average plot of SSTA (°C) with standardized anomalies of a) mean monthly temperature and b) mean monthly rainfall. Red (Blue) filled segments of the SSTA plot that are above (below) 0.5°C (-0.5°C) represent an El Niño (a La Niña) event.

**Fig 4:**
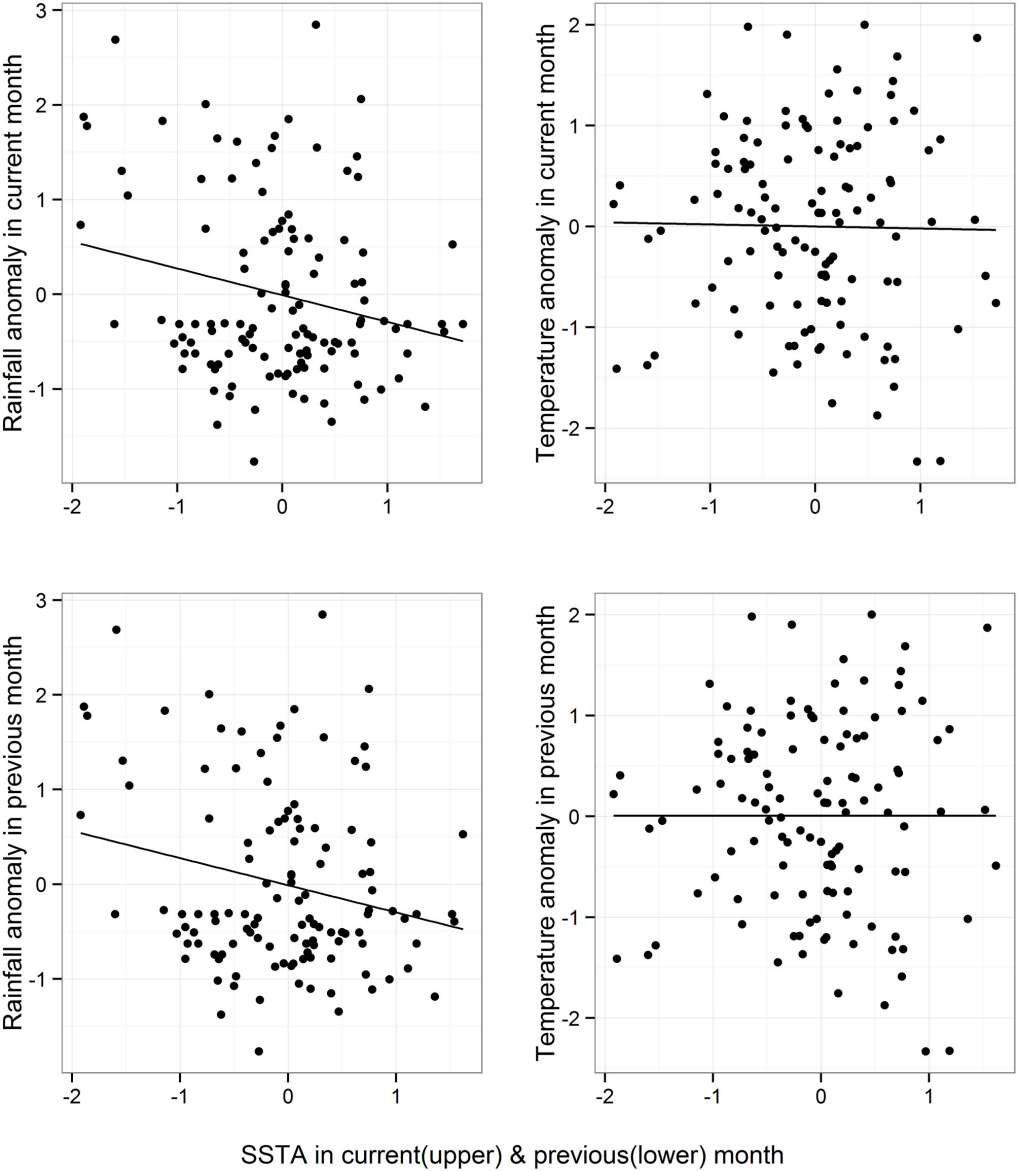
Scatterplot of standardized anomalies of mean monthly rainfall and mean monthly temperature versus SSTA (°C) in current and previous months. The solid line shows the “best-fit” linear regression line.

To investigate how SSTA influences climatic anomalies in Dhaka, standardized anomalies of temperature, relative humidity (S1 Fig), and rainfall were calculated and plotted with SSTA over the study period (Fig 3). Simple linear regression of temperature, relative humidity (S1 Fig), and rainfall anomaly on SSTA at lag of zero and one month revealed a weak negative correlation between rainfall and SSTA (Fig 4) even though the relationship is not temporally consistent (Fig 3) presumably due to a non-linear relationship between them.

Model diagnostics were performed as follows. Firstly, a model was run without the interaction terms and compared with model (1). The likelihood ratio test confirmed that the addition of interaction terms resulted in a significantly improved fit compared to the model without interactions (*p*<0.000). The Pearson dispersion statistic (0.98) also provided evidence for the goodness-of-fit of the model (1). Secondly, residual analyses were performed to ensure that the model provided an adequate fit to the data. Serial autocorrelation of the residuals was checked by examining a time plot and a partial autocorrelation plot of the residuals (S2 and S3 Figs). In addition, observed vs fitted plot of dengue cases was examined (S4 Fig).

## Discussion

It is well established that temperature influences vector and virus biology and therefore dengue transmission. Monthly changes in average temperature have been reported to be positively associated with dengue transmission in Puerto Rico [30]. In addition to average temperature, temperature fluctuations also have an impact. Large fluctuation around warmer temperature reduces transmission whereas around cooler temperature this speeds up the process and vice versa [7, 8, 10]. However, studies of climate and dengue usually ignore diurnal temperature variation. We found that dengue incidence in Dhaka was significantly influenced by mean temperature, DTR, and their synergistic effect, after adjusting for rainfall, anomalies in sea surface temperature, population density, autoregression and the long term temporal trend in dengue incidence. Although mean temperature and DTR were positively associated with dengue incidence, the opposing direction of their interaction term suggested a negative synergy between these two variables. This indicates that although increased mean temperature and reduced DTR or reduced mean temperature and increased DTR increase dengue incidence one month later, an increase or decrease in both lessen dengue incidence. This is consistent with studies showing a positive association between DTR and dengue at low temperatures and a negative association at high temperatures [7, 8, 10]. Use of mean temperature alone in predicting dengue outbreaks will therefore fail to capture the full complexity of the relationship between temperature and dengue transmission.

We demonstrated that increased incidence of dengue in Dhaka was associated with an increase in rainfall in the previous two months. However, an earlier study in Dhaka identified a significant positive association only at lag of two months [22]. The effect of rainfall on *Ae*. *aegypti* breeding is lessened by the species’ egg laying in artificial containers filled with water by humans. But *Ae*. *albopictus* has also been found in Dhaka [31]. Its dependence on rain-fed outdoor artificial containers as larval habitats might explain the positive association between rainfall and dengue incidence. Such a relationship has also been reported in other countries [13, 32]. In Puerto Rico, rainfall has been proposed to have caused increases in dengue incidence by increasing *Ae*. *aegypti* density, egg laying in water storage containers and discarded tires [33].

In Thailand, monthly dengue incidence and epidemics of dengue have been associated with ENSO, which is believed to cause changes in temperature and relative humidity [34]. At time lags of one to 11 months, both epidemics and monthly cases are correlated with El Niño, which is associated with higher temperature and in some places with lower relative humidity [34]. A multivariate ENSO index, lagged at one to six months, alone explains a maximum 22% of the variations in monthly dengue cases [34]. An increase in the number of dengue cases following El Niño was also observed in Mexico, French Guiana, Indonesia, Colombia, and Surinam [13, 18]. The role of ENSO in the inter-annual variability of monsoon rainfall in Bangladesh has been examined demonstrating that El Niño is generally associated with lower rainfall, whereas La Niña and sometimes moderate El Niño generate higher rainfall [35]. However, the relationship is not consistent over time and ENSO is only partially responsible for the rainfall anomalies in Bangladesh. Our study found a negative effect of SSTA on current dengue incidence together with a positive effect at a lag of one month. Possible explanations for the negative association with current SSTA could be that the dry weather resulting from a strong El Niño or the heavy rainfall associated with a moderate El Niño both reduce adult mosquito survival [11, 12] and thereby reduce DENV transmission. Heavy rainfall, on the other hand, could increase transmission because people do not cover themselves in the post-rainfall humid weather resulting in increased human-mosquito contact. The positive effect of SSTA on dengue incidence at a lag of one month is biologically plausible because moderate rainfall is needed for mosquito development, and is also consistent with our findings of a positive influence of rainfall on dengue transmission at a lag of one month. However, heavy rainfall washes away mosquito larvae reducing vector numbers thereby transmission in the following month. Consideration of the non-linear influence of ENSO on rainfall may provide a richer insight into the relationship between dengue and SSTA.

Socio-demographic and economic factors, as well as climate, powerfully influence dengue incidence. A study projects the population at risk of dengue in 2050 under global climate change considering gross domestic product per capita (GDPpc) as an indicator of socio-economic development [19]. The study reports 5.0% and 4.0% increases in the population at risk of dengue in 2050 compared to the baseline risk population in 2000 considering only the projected increase in population and the projected changes in both climate and GDPpc, respectively. Positive but non-significant effects of population growth on dengue cases have also been reported in Mexico [13]. In our study in Dhaka population density was used as a proxy for socio-demographic factors and was found to be positively associated with dengue incidence.

The strength of the present study is that we considered both small and large-scale climatic influences on dengue incidence along with the interaction between mean temperature and DTR and included population density in the model as a proxy for socio-demographic changes over time. However, while we demonstrated significant associations between temperature and rainfall with dengue transmission we did not model non-linear relationships, and we excluded relative humidity from our model due to its strong correlation with mean temperature and DTR. We used months as our temporal unit of study because daily data on dengue incidence were not available. As a consequence, short-scale influences of climatic parameters on dengue incidence may not be fully captured by our model, and lag effects cannot be determined at a fine time-scale. Another limitation of the model used here is that it did not allow for under-reporting from passive surveillance data or possible changes in the rate of under-reporting. However, inclusion of a temporal trend variable in the model may indirectly capture variation in the rate of under-reporting.

In conclusion, our findings indicate that the association between weather and dengue transmission is complex, which is further confounded by socio-demographic factors like population density. Models designed for forecasting should account for this complexity in order to minimize the risk of overestimation in relation to increasing mean temperature, thereby optimizing resource allocation in tropical overpopulated countries with limited resources.

## Supporting Information

S1 Fig a) Three-month moving average plot of SSTA (°C) with standardized anomalies of mean monthly relative humidity. Red (Blue) filled segments of the SSTA plot that are above (below) 0.5°C (-0.5°C) represent an El Niño (a La Niña) event b) Scatterplot of standardized anomalies of mean monthly relative humidity versus SSTA (°C) in current month. The solid line shows the “best-fit” linear regression line.
(TIF)

S2 Fig Partial autocorrelation plot of Pearson residuals. (TIF)

S3 Fig Time series plot of Pearson residuals. (TIFF)

S4 Fig Observed vs fitted plot of dengue cases. (TIFF)
