# Supplementary figures and images for: Interaction of Mean Temperature and Daily Fluctuation Influences Dengue Incidence in Dhaka, Bangladesh
Source: PLoS Negl Trop Dis. 2015 Jul 10;9(7):e0003901. doi: 10.1371/journal.pntd.0003901 (PMC4498835; doi:10.1371/journal.pntd.0003901)

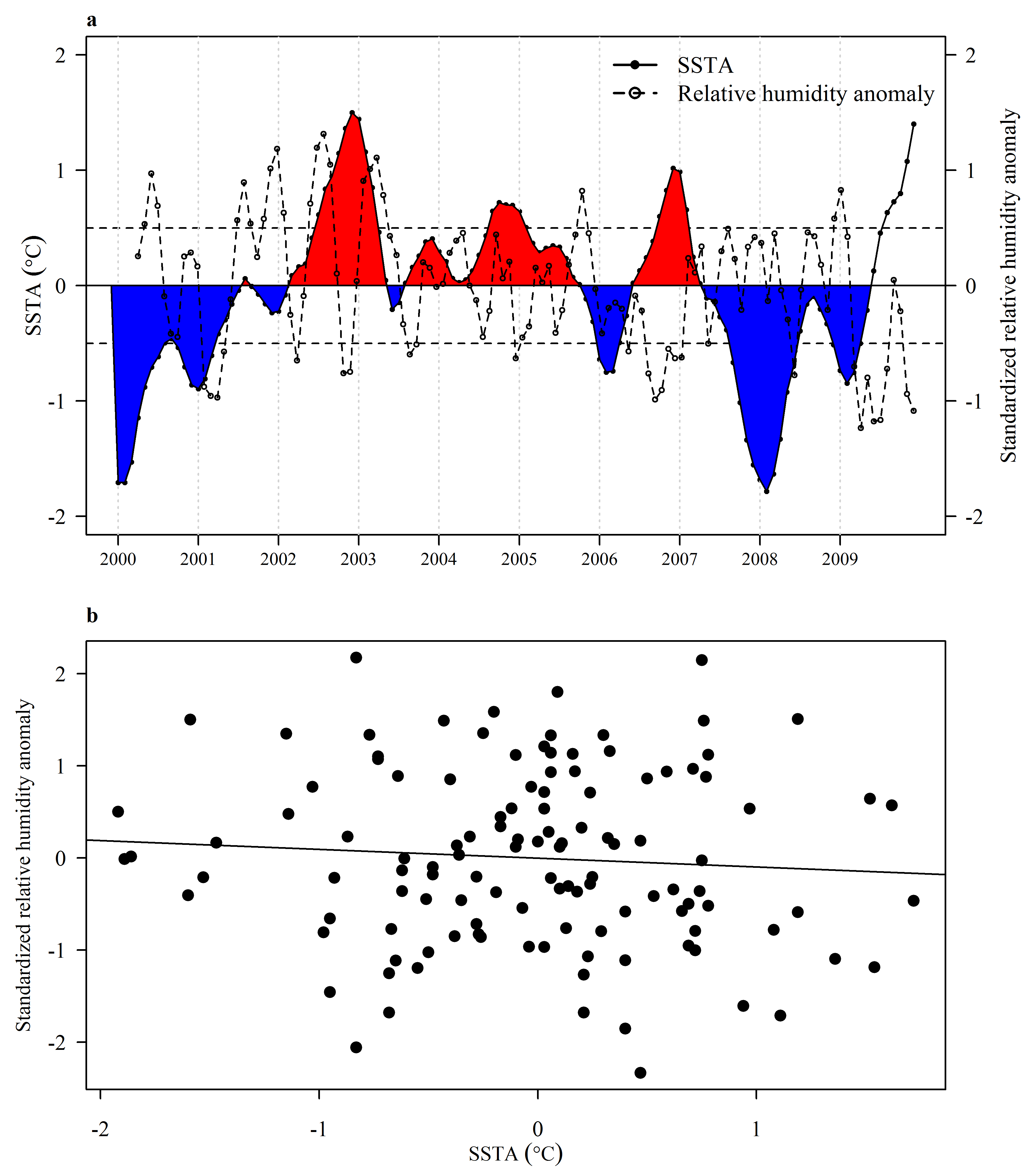

Supplement: S1 Fig — The solid line shows the “best-fit” linear regression line. (TIF) [file pntd.0003901.s001.tif]

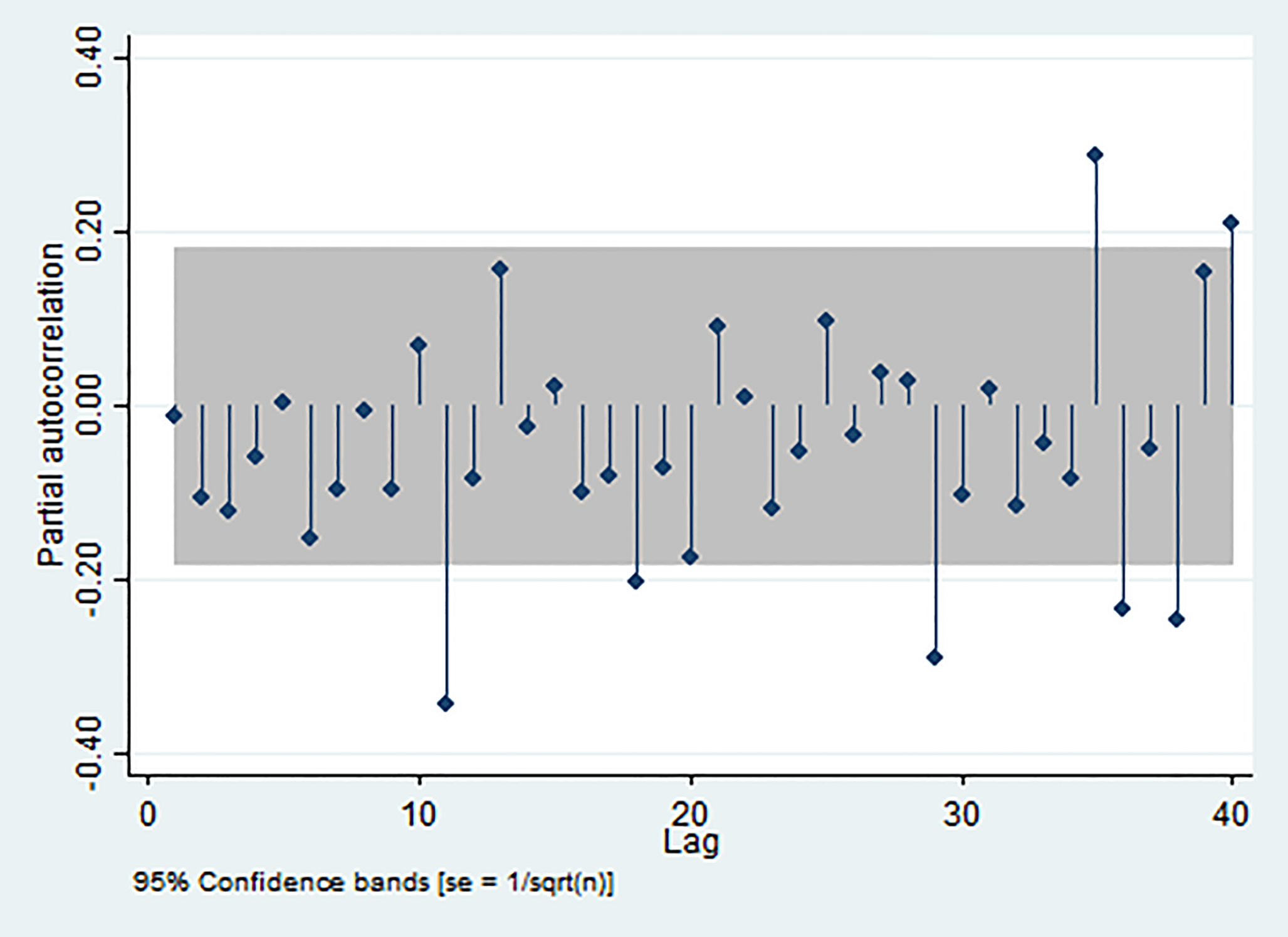

Supplement: S2 Fig — (TIF) [file pntd.0003901.s002.tif]

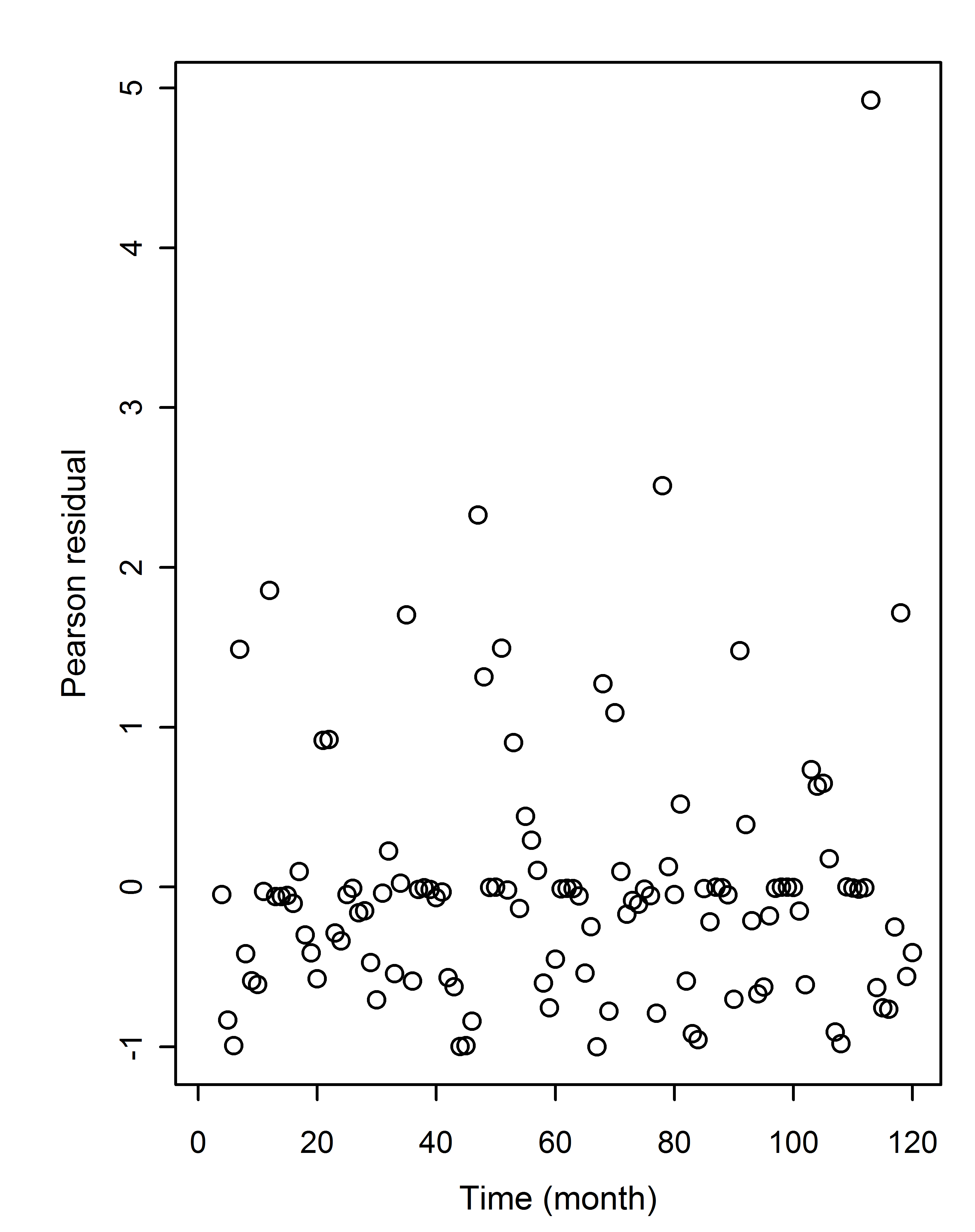

Supplement: S3 Fig — (TIFF) [file pntd.0003901.s003.tiff]

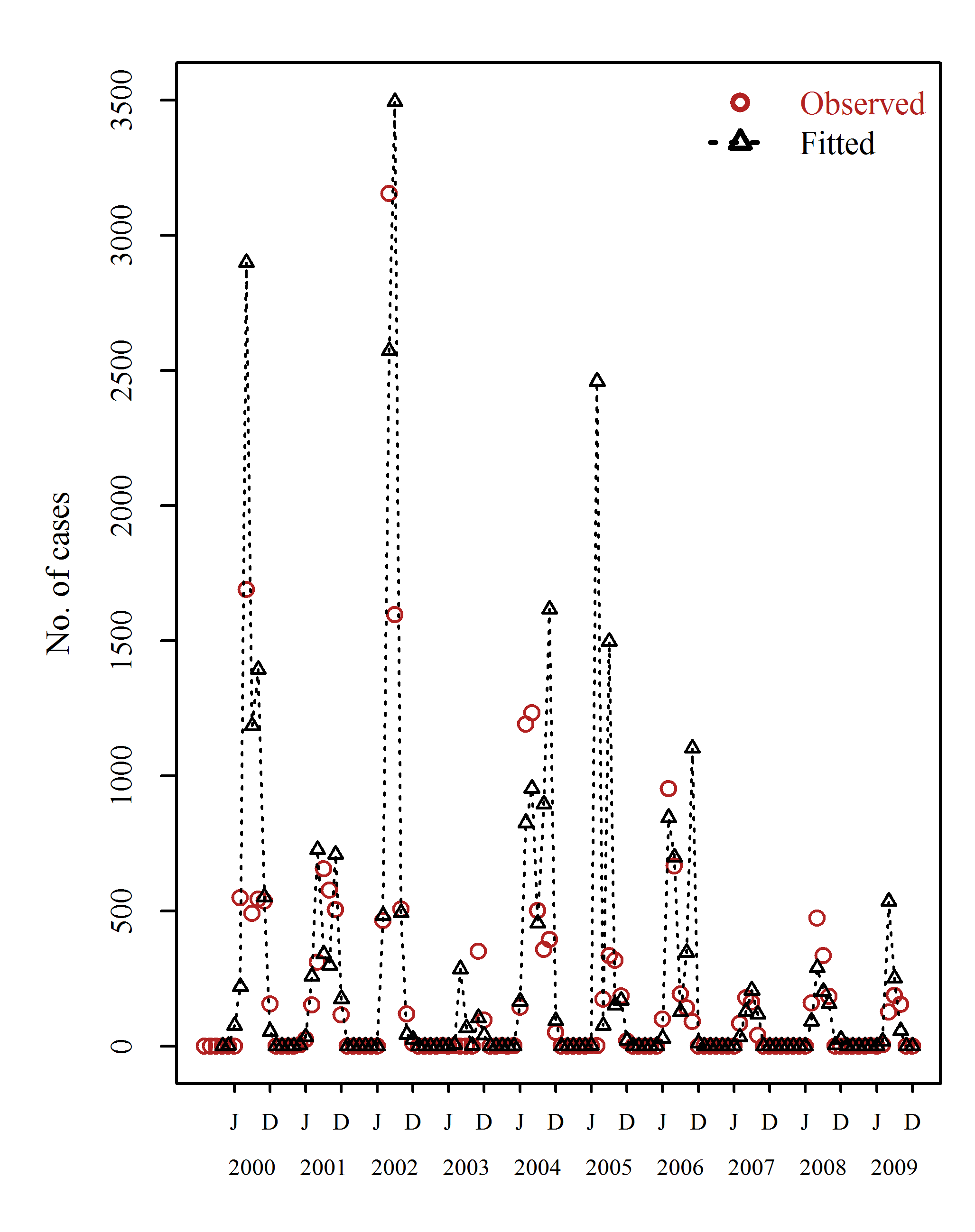

Supplement: S4 Fig — (TIFF) [file pntd.0003901.s004.tiff]
